# Evaluating the Performance of AI Large Language Models in Detecting Pediatric Medication Errors Across Languages: A Comparative Study

**DOI:** 10.3390/jcm15010162

**Published:** 2025-12-25

**Authors:** Rana K. Abu-Farha, Haneen Abuzaid, Jena Alalawneh, Muna Sharaf, Redab Al-Ghawanmeh, Eyad A. Qunaibi

**Affiliations:** 1 Clinical Pharmacy and Therapeutics Department, Faculty of Pharmacy, Applied Science Private University, Amman 11937, Jordan; 2 Department of Pharmacy, Faculty of Pharmacy, Al-Zaytoonah University of Jordan, Amman 11733, Jordan; haneen.abuzaid@zuj.edu.jo; 3 Department of Allied Medical Sciences, Faculty of Applied Medical Sciences, Jordan University of Science and Technology, Irbid 22110, Jordan; jmalalawneh22@ams.just.edu.jo; 4 Department of Pediatric Endocrinology, Faculty of Medicine, AlNajah National University, Nablus P.O. Box 797, Palestine; muna.sharaf@najah.edu; 5 Department of Pediatrics, Faculty of Medicine, The Hashemite University, Zarqa 13133, Jordan; redab@hu.edu.jo; 6 MedOne Academy, 8 The Green, Dover, DE 19901, USA; eyadq@medone.academy

**Keywords:** AI models, accuracy, sensitivity, specificity, reproducibility, pediatrics, medication errors

## Abstract

**Objectives**: This study aimed to evaluate the performance of four AI models, (GPT-5, GPT-4, Microsoft Copilot, and Google Gemini), in detecting medication errors through pediatric case scenarios. **Methods:** A total of 60 pediatric cases were analyzed for the presence of medication errors, of which only half contained errors. The cases covered four therapeutic systems (respiratory, endocrine, neurology, and infectious). The four models were exposed to the cases in both English and Arabic using a unified prompt. The responses for each model were used to calculate various performance metric cover accuracy, sensitivity, specificity and reproducibility. Analysis was carried out using SPSS version 22. **Results:** Microsoft Copilot demonstrated relatively higher accuracy (86.7% in English, 85.0% in Arabic) compared to other models in this dataset, followed by GPT-5 (81.7% in English, 75.0% in Arabic). GPT-4 and Google Gemini had less accuracy, with Gemini having the lowest accuracy across all languages (76.7% in English, and 73.3% in Arabic). Microsoft Copilot showed comparatively higher sensitivity and specificity, particularly in cases of respiratory and infectious diseases. The accuracy in Arabic was lower compared to that of English for the majority of models. Microsoft Copilot exhibited relatively higher reproducibility and inter-run agreement (Cohen’s Kappa = 0.836 English, 0.815 Arabic, *p* < 0.001 for both), while Gemini showed the lowest reproducibility. For inter-language agreement in general, Copilot showed the highest Cohen’s Kappa of 0.701 for English and Arabic (*p* < 0.001). **Conclusions:** In our evaluation, Microsoft Copilot demonstrated relatively higher performance in pediatric drug error detection compared to the other AI models. The decreased performance in Arabic points toward the requirement of improved multilingual training for supporting equal AI aid across languages. This study highlights the importance of human oversight and domain-based training for AI tools in pediatric pharmacotherapy.

## 1. Introduction

In the past decade, there has been a clear surge in research focused on artificial intelligence (AI), machine learning (ML), and their algorithms within healthcare and medical systems [1,2]. Innovations in AI have driven major advances in patient triage, diagnosis, personalized treatment planning, and ongoing monitoring [3,4], which in turn have contributed to improved patient outcomes [5]. However, it is important to understand that AI systems do not think or reason like humans [6,7]; instead, they process large amounts of data using sophisticated algorithms to detect patterns [8]. Consequently, the accuracy of their outputs depends largely on the quality and diversity of the data used to train them [9].

Pediatric care, which requires exceptional accuracy, has seen valuable contributions from AI technologies [10,11,12]. These technologies assist healthcare providers by calculating medication doses based on individual factors such as body weight and organ function, thereby minimizing errors [13,14,15]. Additionally, AI tools have enhanced early detection of developmental issues by analyzing growth and vital signs data, enabling timely interventions [16]. These advancements have strengthened clinical decisions and improved care quality for children.

Despite progress, the ability of AI to detect potential errors in pediatric medication dosing or inappropriate prescriptions remains insufficiently studied. This issue is critical because many drugs lack pediatric approval, increasing the risk of errors [17]. Furthermore, since most AI tools are primarily trained in English [18], the predominant global language, their performance in other languages, such as Arabic, remains uncertain. This is a notable concern, given the large number of Arabic speakers worldwide.

Thus, this study aimed to address this gap by comparing the performance of four AI models, one subscription-based (GPT-5) and three freely accessible models (GPT-4, Microsoft Copilot, and Google Gemini), in detecting medication errors across pediatric case scenarios developed in two languages (English and Arabic). The study primarily focused on the models’ accuracy, sensitivity, specificity, reproducibility, and response agreement between languages.

## 2. Materials and Methods

### 2.1. Study Design and Case Development

This study represents a diagnostic comparative study that aimed to evaluate the performance of four AI large language-based models in detecting medication errors among pediatric case scenarios. The study took place between July and August 2025.

In this study 60 case scenarios were developed by the research team which consists of three PhD holder pharmacists (two of whom are full professors), a pediatric neurologist, a pediatric endocrinologist and a respiratory specialist (Supplementary Table S1). Only half of the cases contain one type of errors, while the remaining were intentionally error-free. This was performed to assess the ability of AI tools to detect the presence or absence of errors, allowing for the evaluation of their sensitivity and specificity. Errors were classified into three types as follows: 12 incorrect dosing, 8 incorrect frequencies and 10 incorrect drug selection. The cases reflect four main therapeutic domains including respiratory, endocrine, neurology, and infectious. Each domain contains 15 cases.

The case scenarios were developed in accordance with the lead authorities’ reference that include textbooks including Harriet Lane Handbook, the Lexicomp Pediatric & Neonatal Dosage Handbook, the Pediatrics Review and Education Program. Also, the recent updated clinical guidelines and up-to-date were used. These cases do not reflect real-world complexity. The cases were designed to be idealized, with complete and accurate information, each containing only one error. This approach was used to assess the AI models’ ability to detect a single error in a controlled, simplified environment.

Before evaluating the performance of the selected AI tools, the cases were first validated for accuracy by four pediatric specialists, each chosen for their expertise in one of the four therapeutic domains. Each specialist reviewed 15 cases specific to their area of expertise: the pediatric respiratory cases were assessed by a pediatric respiratory specialist, the endocrine cases by a pediatric endocrinologist, and so on. The specialists were aware of the study’s aims and were instructed not to use any AI tools while performing their reviews. The specialists’ feedback was then reviewed by the research team, who cross-referenced it with established guidelines. If necessary, the cases were revised accordingly to ensure accuracy, as shown in Table 1.

For the purpose of the study, to enable bilingual assessment of each AI tool, the cases were translated into Arabic by two colleagues with expertise in the medical domain, both fluent in English and Arabic. One researcher performed the initial translation from English to Arabic, while the second conducted the back-translation from Arabic to English. The back-translated version was then compared to the original English version by the research team to ensure that the translation accurately preserved the clinical meaning and intent. No significant discrepancies were identified during this process, and the translation was considered clinically equivalent for the purpose of the study.

### 2.2. AI Tools for Evaluation

Four large language model-based tools were selected for evaluation. These included one paid subscription model, GPT-5 (released on 7 August 2025), and three freely accessible tools: GPT-4o (released on 13 May 2024), Google Gemini 2.5 Flash (released on 17 June 2025), and Microsoft Copilot in Bing (released in February 2023, upgraded to GPT-5 in August 2025). The selection aimed to cover a range of models available to both paying users and the general public. All models were accessed via their respective web interfaces during the evaluation process. For GPT-5, access was via the subscription-based interface at chat.openai.com, while the others were accessed via web interfaces without any login requirements. All models were set with their default configurations, and memory was disabled with a new chat initiated for each case to ensure independence of evaluations.

### 2.3. Prompting Procedure and Data Collection

To evaluate the performance of the AI tools, the same prompt was used to ensure consistency in providing instructions. Also, the “new chat” interface, along with memory disabling, was used for each interaction to avoid any influence from previous interactions.

The English prompt used for all interaction was “Please review the following pediatric case and determine whether a medication error is present or not. If present, please identify and explain it.” The equivalent Arabic prompt was “يرجى مراجعة الحالة الطبية التالية لطفل وتحديد ما إذا كان هناك خطأ دوائي. إذا وُجد خطأ، يُرجى تحديده وشرحه”.

All AI-generated responses were documented with screenshots and organized for subsequent analysis. Each model evaluated all 60 cases in both English and Arabic, producing a total of 480 initial responses (4 models × 2 languages × 60 cases). The evaluation of all models was conducted concurrently over a 3-day period, from 14 to 16 August 2025, with each tool’s evaluation completed within 24 h.

### 2.4. Classification of Responses

Each AI response was evaluated based on its ability to correctly identify the presence or absence of medication errors. The responses were categorized by the research team as follows: True Positive (TP), where the AI tool correctly detects a real medication error; True Negative (TN), where the AI tool correctly identified the absence of a real medication error; False Positive (FP), which are cases where the AI tool detects an error when there is actually no error; and False Negative (FN), which occurs when the tool specifies that no error is detected while actually there was an error, or if it detects an incorrect error.

### 2.5. Reproducibility Testing

To evaluate AI tools consistency in predicting errors over time, the same evaluation process was repeated for a second round after two weeks, from 28 to 30 August 2025, for all tools in both languages using the same prompt. Consistency was assessed using reproducibility index, which represents the proportion of identical responses across the two runs. Also, the agreement between the two runs was evaluated using Cohen’s Kappa (κ) Statistic.

For reproducibility scoring, the responses from the first and second runs were assessed separately. Following independent evaluations of both runs, discrepancies between the two were identified. In cases of disagreement, these were manually adjudicated by the research team to ensure consistency and resolve any ambiguities.

### 2.6. Performance Metrics

To evaluate the performance of the selected models, several metrics were calculated as follows:

Accuracy: which represents the proportion of correctly identified responses (both TP and TN) out of the total responses.

[Accuracy = (TP + TN)/(TP + TN + FP + FN)]

Sensitivity: The proportion of medication error identified (TP) out the total number of cases containing errors (TP + FN).

[Sensitivity = TP/(TP + FN)]

Specificity: The ability of the model to correctly identify error-free cases (TN) out the total number of error-free cases (TN + FP).

[Specificity = TN/(TN + FP)]

Reproducibility index: The proportion of identical responses across the two runs.

Cohen Kappa: To measure the agreement between the two languages for each model, as well as the agreement between the first and second runs of each model.

### 2.7. Statistical Analysis

Data were coded and entered to IBM SPSS Statistical Software (version 22). Descriptive statistics were presented using frequency and percentages for categorical variables, while mean ± standard deviation were used for continuous variables. Normality was checked using the Shapiro–Wilk test, with *p* > 0.05 indicating normally distributed variable. Homogeneity of variances was tested using Levene’s test. Each model underwent four runs: the first run in both English and Arabic, and the second run in both English and Arabic. The means of these four runs for each model were compared using One-way Analysis of Variance (ANOVA). When ANOVA yielded a significant result, Tukey’s Honestly Significant Difference (HSD) test was used for post hoc pairwise comparisons.

The agreement between AI models was assessed using Cohen’s Kappa coefficient, with pairwise comparisons conducted using a Bonferroni correction, resulting in a significance threshold of *p* ≤ 0.0083. Statistical significance was set at a threshold of *p* ≤ 0.05.

## 3. Results

In this study, 60 pediatric cases were included, among them 30 (50.0%) were error-free. In evaluating the confusion matrix, Microsoft Copilot achieved a relatively higher number of correct answers among the tested models in both languages, with 52 correct in English (29 TP, 23 TN) and 51 correct in Arabic (28 TP, 23 TN). This was followed by GPT-5 in English, with 49 correct (27 TP, 22 TN). Google Gemini demonstrated comparatively lower performance within this dataset, with 46 correct in English (29 TP, 19 TN) and 44 correct in Arabic (23 TP, 21 TN).

**Figure 1:**
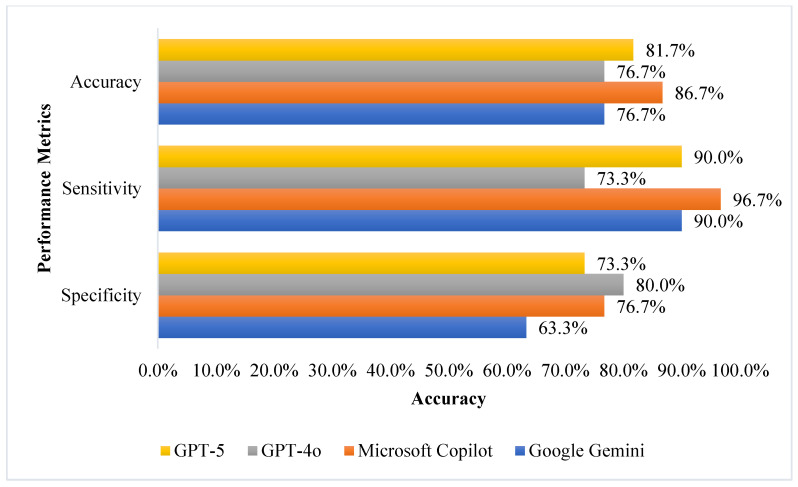
AI Models Performance Summary in Predicting Medication Errors (Accuracy, Sensitivity, and Specificity) in English. The values represent single-point estimates of accuracy, sensitivity, and specificity from the first run for each model, conducted in English.

**Figure 2:**
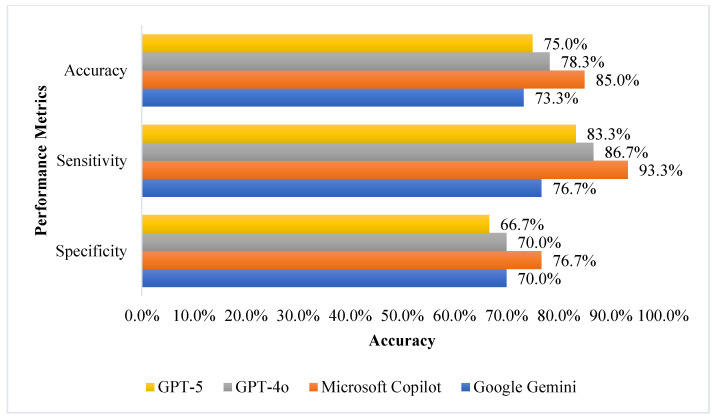
AI Models Performance Summary in Predicting Medication Errors (Accuracy, Sensitivity, and Specificity) in Arabic. The values represent single-point estimates of accuracy, sensitivity, and specificity from the first run for each model, conducted in Arabic.

Accuracy, sensitivity, and specificity for all tools are shown in Figure 1 (English) and Figure 2 (Arabic) demonstrating that Microsoft Copilot showed relatively higher accuracy (86.7% in English and 85.0% in Arabic) compared to other models in this sample, and sensitivity above 93% in both languages. GPT-5 achieved an accuracy of 81.7% and sensitivity of 90.0% in English, but its performance decreased in Arabic (accuracy 75.0%, sensitivity 83.3%). GPT-4o showed improvement in both accuracy and sensitivity when moving from English (accuracy 76.7%, sensitivity 73.3%) to Arabic (accuracy 78.3%, sensitivity 86.7%). Google Gemini had the lowest accuracy and sensitivity in Arabic (73.3%).

Specificity was highest for GPT-4o in English (80.0%) and remained consistent for Copilot in both languages (76.7%), while the lowest specificity was observed for GPT-5 in Arabic (66.7%) and for Gemini in English (63.3%).

Table 2 showcases examples of the top 3 and lowest 3 correctly answered questions by the four AI models. Out of 60 total questions, 23 were answered correctly by all four AI tools in both languages (English and Arabic), demonstrating agreement of AI performance in certain medical scenarios. However, the remaining questions showed varying levels of accuracy, with some, particularly in respiratory and infectious diseases, receiving as few as 1–3 out of 8 correct responses.

When comparing AI models across therapeutic systems (Table 3), in the respiratory system, Microsoft Copilot achieved 86.7% accuracy in English versus 73.3% in Arabic, with sensitivity remaining at 100% for both languages, and specificity decreasing from 77.8% to 66.7%. GPT-5 showed a similar trend, dropping from 60.0% accuracy in English to 53.3% in Arabic, and sensitivity from 66.7% to 33.3%. Google Gemini performed better in Arabic (86.7% accuracy) compared to English (66.7%), though specificity varied widely (100% vs. 44.4%).

For the endocrine system, performance was largely consistent across languages for most models. Copilot, GPT-5, and Gemini maintained identical results in English and Arabic: 86.7% accuracy, 100% sensitivity, and 71.4% specificity. GPT-4o showed a slight difference, with English accuracy at 73.3% versus 86.7% in Arabic, and specificity improving from 57.1% to 71.4%.

In the neurology domain, GPT-5 demonstrated higher accuracy in English (93.3%) compared to Arabic (80.0%), with specificity dropping from 100% to 83.3%. Copilot showed a modest decline from 86.7% to 80.0% accuracy, while sensitivity remained relatively stable (87.5% vs. 100%). Gemini exhibited similar performance in both languages (80.0% accuracy).

Finally, in infectious diseases, Copilot achieved the highest accuracy in English (93.3%) compared to 66.7% in Arabic, with specificity decreasing sharply from 85.7% to 42.9%. GPT-5 maintained strong performance in both languages (86.7% English vs. 80.0% Arabic), while GPT-4o and Gemini showed moderate declines in Arabic.

When assessing the performance between the four AI tools (Table 4), a significant difference in accuracy was observed, with Microsoft Copilot demonstrated higher mean accuracy (85.5 ± 1.7) than Gemini (73.3 ± 2.7) within this dataset, with a *p*-value of 0.005. Also, GPT-5 (83.4 ± 7.0) outperforms Gemini in accuracy, with a *p*-value of 0.017. Even though ANOVA testing revealed statistically significant differences between tools in their sensitivity, post hoc analysis did not reveal any significant in the pairwise comparison. Similarly, ANOVA testing did not show significant differences between tools in terms of their specificity.

Table 5 compares the reproducibility and agreement of AI models between their first and second runs across English and Arabic. Microsoft Copilot shows the highest reproducibility (90.0% in English, 88.3% in Arabic) and strongest agreement (Cohen’s Kappa: 0.836 and 0.815, respectively). GPT-5 showed relatively strong reproducibility in English (86.6%). Cohen’s Kappa = 0.783), while Google Gemini shows lower reproducibility and agreement both in English and Arabic (70.0% reproducibility, Cohen’s Kappa ≈ 0.57).

When comparing the reproducibility and inter-language agreement of AI models between English and Arabic during the first run (Table 6). Microsoft Copilot exhibited relatively higher reproducibility (81.7% identical outputs) and agreement (Cohen’s Kappa = 0.701) among the tested models in this dataset. GPT-5 and Google Gemini follow with reproducibility indices of 76.7% and 73.3%, and Kappa values of 0.650 and 0.610, respectively. GPT-4o has the lowest reproducibility (68.3%) and agreement (Cohen’s Kappa = 0.532).

## 4. Discussion

To our knowledge, this is the first comparative bilingual evaluation of AI models in pediatric pharmacotherapy, assessing their accuracy, sensitivity, specificity and reproducibility in detecting medication errors. The four evaluated models (GPT-5, GPT-4o, Microsoft Copilot, and Google Gemini) achieved accuracies ranging from 53.3% to 93.3% in detecting pediatric medication errors across respiratory, endocrine, neurological, and infectious disease systems. The evaluation encompassed identifying errors in dosing regimen and drug selection. Notably, only 23 of our 60 prescriptions (38.3%) were correctly answered by all four AI tools in both languages. This indicates that although individual accuracies exceed 50%, inter-model concordance on individual cases remains limited, highlighting the importance of continued human oversight in pediatric prescribing.

In our evaluation, GPT-4o achieved 76.7% accuracy in English, highlighting that the previously reported 100% accuracy of GPT-4o in pediatric dosing tasks [19] should be interpreted cautiously, as it does not generalize across all pediatric prescription domains.

Also, in our dataset, Microsoft Copilot showed relatively higher accuracy compared to GPT-5 and GPT-4o models, and all demonstrated higher accuracy than Gemini within the evaluated cases. Such variation in how AI models perform in medication-related and pediatric contexts has been reported previously. For instance, Claude 3.5 Sonnet and GPT-based models achieved comparatively higher performance than Gemini models in detecting and correcting prescribing errors [20]. Moreover, Microsoft Copilot provided a higher proportion of correct pediatric ophthalmology answers than GPT and Google Gemini (*p* < 0.05) [21]. However, this lower performance of Google Gemini is not generalizable; when evaluated on pediatric orthopedics questions, GPT and Google Gemini achieved higher answer accuracy and completeness compared to Microsoft Copilot (*p* < 0.05) [22].

When the performance of the evaluated AI models was compared across therapeutic systems, the combined mean accuracy for the four models was lowest for respiratory system cases in both English and Arabic. This may reflect either higher case difficulty in the respiratory domain compared with other systems or technical limitations inherent to the evaluated models. The observation that Microsoft Copilot maintained accuracy in respiratory cases comparable to its performance in the other systems may suggest the latter interpretation, though this should be interpreted cautiously given the limited dataset. When sensitivities and specificities are compared, differences were observed across systems; Google Gemini has almost double specificity in neurology compared with respiratory, and GPT-5 has triple sensitivity in endocrine compared with respiratory English cases. Although we did not evaluate AI performance variability based on disease prevalence within the same system, this question has been addressed in the literature. For example, Wei et al., (2023) reported that ChatGPT-4 performed less accurately when providing treatment recommendations for rare pediatric disorders than for common ones [23].

In addition to performance variation resulting from differences in AI models and therapeutic systems, difference in reported accuracies across studies is also brought about by difference in methods of evaluation and case complexity. In this study, we used well-defined single-error pediatric cases to enable a uniform assessment of the ability of each model to detect error, resulting in relatively high scores of accuracies. To compare, Ong et al. (2025) [20] evaluated multi-error cases across multiple therapeutic areas, requiring the detection and correction of concomitant prescribing errors, resulting in decreased levels of accuracy (16% for Gemini Flash to 51% for Claude 3.5 Sonnet). Similarly, Triplett et al. (2025) put the older GPT-3.5 model to the test using drug-information questions, based on a multi-item rubric with rigorous scoring that treated answers having any factual deviation as incorrect, resulting in an overall accuracy of only 50% [24]. Task specificity also influences performance, as demonstrated by Wei et al. (2023), who demonstrated lower accuracy for ChatGPT in generating elaborate pediatric treatment plans or dosage recommendations than for general therapeutic recommendations [23]. These methodological differences explain the variability found in AI accuracy studies related to pharmacotherapy and highlight the importance of careful interpretation of reported scores. In addition, high accuracy figures achieved in controlled or simulated settings -like our study and Levin et al. (2025) [19] of pediatric dosing- may decrease in real-life scenarios where information is missing and prescriptions are written in unclear or inconsistent ways.

Beyond pediatric therapeutics, similar patterns of limited performance hold for pediatric diagnosis use cases. Barile et al. (2024) reported that ChatGPT-3.5 correctly diagnosed only 17 of 100 pediatric cases from JAMA Pediatrics and NEJM case challenges [25].

These findings underscore the importance of human oversight and validation of AI outputs in pediatrics, as models not specifically tuned for children may have limitations that warrant caution [26].

One novelty of our research is the assessment of sensitivity and specificity of AI models in pediatric therapeutics; measures that are rarely reported in this field, although they appear in research on pediatric diagnosis with AI support [27]. Decomposing each model accuracy into sensitivity and specificity provides clinically useful information: low specificity may result in excessive false alerts, which may reduce clinician productivity and contribute to alarm fatigue [28], whereas low sensitivity may result in genuine medication errors being missed. Microsoft Copilot demonstrated relatively higher sensitivity and specificity in most cases for both Arabic and English, except for specificity in English, where GPT-4o showed slightly higher performance.

In our study, AI models showed lower accuracy in detecting errors in Arabic cases compared to their English counterparts across most therapeutic systems, with the largest divergence noted in infectious diseases (76.7% vs. 83.3%). Sallam et al. (2024) also found that AI models responded consistently better to infectious disease cases in English compared to Arabic in four tested models (Bard, Bing, ChatGPT-4, and ChatGPT-3.5) [29]. This language variation in performance could be due to biased training data and has significant implications, potentially compromising AI-generated health information for clinicians in resource-limited settings who rely on Arabic, and who stand most to gain from accurate AI support in pediatric prescribing. This variation points to the need for AI developers to fill these gaps to promote equitable global adoption. In our dataset, Microsoft Copilot showed relatively higher performance across most metrics for detecting errors in Arabic cases, followed by GPT-4o, while GPT-5 performed slightly below GPT-4o.

Other than differences in language, a sector that needs improvement is the level of clinical training itself. The results of our study emphasize the importance of domain-specific training in order to improve AI performance in pediatrics. A recent study using Italian pediatric questions showed that training AI models on datasets that capture the complexities of pediatric conditions was associated with improved accuracy in answering pediatric questions [30].

To be reliable, AI responses need to be not only accurate but also consistent across runs [31]. Our evaluation of reproducibility and agreement across the AI models over two runs (2 weeks apart) showed a similar pattern to their accuracy: Microsoft Copilot demonstrated relatively higher reproducibility and agreement, while Google Gemini showed comparatively lower performance in both English and Arabic. All Cohen’s κ values were highly significant (*p* < 0.001), indicating agreement well beyond chance. This reproducibility suggests potential clinical relevance, as it indicates Microsoft Copilot’s performance was not only accurate but also consistent across repeated tests. On the other hand, the moderate κ of Gemini warrants some caution for that model. We observed high reproducibility (78.3%) and agreement (κ = 0.676) for GPT-4o compared to Khatri et al., who reported a κ of only 0.23 between runs 1 and 2 for GPT-4 responding to real-world drug-information queries [31]. This difference likely stems from the design of their research, in which they challenged GPT-4 with open-ended drug-information questions requiring narrative answers that were scored by a strict rubric, compared with our standardized, binary pediatric cases.

In comparing reproducibility and inter-language agreement between English and Arabic in the first run, Microsoft Copilot showed relatively higher reproducibility compared to other models in this dataset. Since its high reproducibility was accompanied by high accuracy across languages, this combination indicates reliable performance. In contrast, GPT-4o lagged behind GPT-5 and Gemini, showing lower reproducibility between its English and Arabic outputs despite similar accuracy, indicating less stability when shifting to Arabic.

## 5. Conclusions

By systematically comparing widely used AI platforms in pediatric pharmacotherapy, we identified notable differences in how effectively they detected medication errors across languages and therapeutic systems. In our evaluation, Microsoft Copilot demonstrated relatively higher consistency and accuracy, while Google Gemini showed comparatively lower accuracy and less stable results. The observed better performance in English compared with Arabic suggests a need for balanced training to ensure equitable AI support for clinicians who use AI in Arabic. Measuring sensitivity, specificity, and reproducibility to added value in understanding the clinical reliability of AI tools beyond overall accuracy. These findings show both the potential and current limitations of AI in pediatric pharmacotherapy and suggest the importance of domain-specific training, multilingual optimization, and vigilant human oversight during clinical use.

## 6. Limitations

This study was performed using 60 pediatric cases, half of which contained a single medication error, while the remaining cases were intentionally error-free. Real-world performance might differ due to concurrent errors, handwriting-related errors, and errors related to incomplete notes. Also, since this study included only single-error cases, this may inflate accuracy relative to real-world scenarios, where multiple errors are more likely to occur concurrently. Moreover, the accuracy of cases across pediatric subspecialties that were not represented here might vary. Thus, real-world multi-specialty studies are needed to improve generalizability. Additionally, the assessment of the prevalence and the difficulty of the evaluated pediatric cases was not covered in our study. In addition, the relatively small sample size of 60 cases (30 error-containing and 30 error-free) limits statistical power for more granular subgroup analyses, particularly across the four therapeutic systems and two languages. Larger sample sizes in future studies would improve the power for such analyses.

## 7. Future Directions

Future work can explore how the integration of large language models (LLMs) into health system workflows for checking pediatric prescriptions and detecting possible errors can affect clinical outcomes. As previously shown, such integration proves its advantage in improving pharmacist detection of errors in prescriptions [20]. Moreover, expanding the evaluation of AI models to encompass multilingual datasets and additional pediatric subspecialties will consolidate generalizability. The influence of the prompt is another area to be investigated as engineered and fine-tuned cases could influence models’ accuracy.

## Figures and Tables

**Table 1:** Summary of Pediatric Clinical Case Revisions Based on Specialist Review.

Reviewer Specialty	Cases Reviewed	Revisions Suggested	Actions Taken
Respiratory	1–15	Case 8: First-line treatment for bacterial pneumonia is amoxicillin, not ampicillin.	Corrected to amoxicillin.
		Case 9: Dose calculation exceeds per-dose max (should not exceed 500 mg/dose).	Not changed—exceeding 500 mg/dose is acceptable.
		Case 10: Xopenex (Levalbuterol) is not available.	Not changed—may still be used in other countries.
Endocrinology	16–30	Case 16: Recommend stating that serum thyroid function tests confirmed CH.	Corrected.
		Case 17: Replace “insulin pens” with “multiple daily injections”.	Corrected.
		Case 22: Add frequency of medications (OD, BID, TID).	Corrected.
		Case 23: Add weight and height.	Corrected.
		Case 26: Mention the starting dose.	Corrected.
Neurology	31–45	Case 35: Use generic name “sodium valproate” instead of “Depakine”.	Not changed—brand name retained for AI recognition.
		Case 41: Use generic name “vigabatrin” instead of “Sabril^®^”.	Not changed—brand name retained for AI recognition.
		Case 43: Use “physical examination” and clarify “bid” as “twice daily.”	Corrected.
Infectious Diseases	46–60	Case 47: Urinalysis is suggestive, not confirmatory of UTI. TMP dose is 8 mg/kg/day (not 5), divided BID.	Corrected.
		Case 51: Clarify that this refers to non-typhi Salmonella, as S. typhi has a different treatment.	Corrected.
		Case 52: Described case is early localized Lyme disease, not early disseminated.	Corrected.
		Case 54: Drug of choice for strep throat is penicillin V or amoxicillin, as Group A strep is most likely.	Corrected.
		Case 55: Theophylline is rarely used in pediatric asthma.	Not changed—still used in select cases.
		Case 58: Azithromycin is not recommended for treating or preventing aspiration pneumonia. Use clindamycin instead.	Corrected—Clindamycin used instead.

**Table 2:** Examples of the Top and Lowest Correctly Answered Questions by the Four AI Models in Both Languages (4 × 2 = 8 responses) for the First Run.

Case Number	System	Question	Key Answer	Number of Correct Responses (Out of 8)
21	Endocrine	A 6-year-old girl (22 kg) diagnosed with central precocious puberty, with height on the 95th percentile and advanced bone age, was started on Decapeptyl IM 3.75 mg (triptorelin) every 6 months.	Decapeptyl 3.75 mg should be given at an interval of 28 days or less.	8
32	Neurology	A 10-year-old boy was diagnosed with ADHD. Past medical history: episodes of fainting during exercise. Blood pressure was normal for age. ECG: Shows prolonged QT interval (QTc = 500 ms). He was diagnosed with ADHD and prolonged Q-T syndrome. Prescribed Ritalin 10 mg/day. In two divided doses before breakfast and before lunch	Methylphenidate (a stimulant). And other stimulants can increase heart rate and QT interval, raising the risk of torsades de pointes and sudden cardiac death in patients with Long QT syndrome. Clonidine (alph-2 agonist) is a safe choice	8
52	Infectious	A 14-year-old boy (60 kg) was diagnosed with Lyme disease (early localized, erythema migrans with mild systemic symptoms). He was prescribed doxycycline 100 mg orally once daily for 14 days.	Doxycycline should be 100 mg twice daily for Lyme disease in this age group.	8
13	Respiratory	A 13-year-old girl (45 kg) with fatigue, intermittent fever, and a persistent productive cough. Diagnosed with pulmonary tuberculosis confirmed by sputum. Started on a four-month regimen: daily isoniazid 300 mg, rifapentine 1200 mg, moxifloxacin 400 mg, and pyrazinamide 1500 mg.	Error free	1
51	Infectious	A 9-year-old girl (30 kg) with recent travel history developed bloody diarrhea, abdominal cramps, and fever. Stool culture was positive for Salmonella non-typhi strains. She was prescribed azithromycin 300 mg orally once daily for day 1 then 150 mg for days 2–3.	Error free	2
49	Infectious	A 3-year-old boy (14 kg) with a history of recurrent skin infections presented with a localized cellulitis. He was prescribed clindamycin 150 mg orally every 8 h.	Error free	3

**Table 3:** AI Models Performance Summary in Predicting Medication Errors Across Therapeutic Systems.

	AI Tool	Language	Accuracy	Sensitivity	Specificity
Respiratory	GPT-5	English	60.0%	66.7%	55.6%
		Arabic	53.3%	33.3%	66.7%
	GPT-4o	English	73.3%	83.3%	66.7%
		Arabic	73.3%	83.3%	66.7%
	Microsoft Copilot	English	86.7%	100.0%	77.8%
		Arabic	86.7%	66.7%	100.0%
	Google Gemini	English	66.7%	100.0%	44.4%
		Arabic	60.0%	50.0%	66.7%
Endocrine	GPT-5	English	86.7%	100.0%	71.4%
		Arabic	86.7%	100.0%	71.4%
	GPT-4o	English	73.3%	87.5%	57.1%
		Arabic	86.7%	100.0%	71.4%
	Microsoft Copilot	English	86.7%	100.0%	71.4%
		Arabic	86.7%	100.0%	71.4%
	Google Gemini	English	86.7%	100.0%	71.4%
		Arabic	80.0%	87.5%	71.4%
Neurology	GPT-5	English	93.3%	87.5%	100.0%
		Arabic	80.0%	77.8%	83.3%
	GPT-4o	English	80.0%	62.5%	100.0%
		Arabic	86.7%	75.0%	100.0%
	Microsoft Copilot	English	80.0%	87.5%	71.4%
		Arabic	80.0%	100.0%	57.1%
	Google Gemini	English	80.0%	75.0%	85.7%
		Arabic	80.0%	75.0%	85.7%
Infectious	GPT-5	English	86.7%	100.0%	71.4%
		Arabic	80.0%	88.9%	66.7%
	GPT-4o	English	80.0%	62.5%	100.0%
		Arabic	66.7%	87.5%	42.9%
	Microsoft Copilot	English	93.3%	100.0%	85.7%
		Arabic	86.7%	100.0%	71.4%
	Google Gemini	English	73.3%	87.5%	57.1%
		Arabic	73.3%	87.5%	57.1%

**Table 4:** Evaluating the Differences Between AI Tools in Their Performance Metrics.

Metric	Model	Mean ± SD	95% CI for Mean	*p* Value #
Accuracy	GPT-4o	78.8 ± 2.1	75.4–82.1	0.005
	GPT-5	83.4 ± 7.0	72.3–94.4	
	Microsoft Copilot	85.5 ± 1.7	82.8–88.1	
	Google Gemini	73.3 ± 2.7	69.0–77.7	
Sensitivity	GPT-4o	83.3 ± 8.6	69.6–97.0	0.037
	GPT-5	91.7 ± 6.4	81.5–100.0 *	
	Microsoft Copilot	92.5 ± 4.2	85.8–99.2	
	Google Gemini	77.5 ± 8.8	63.5–91.5	
Specificity	GPT-4o	74.2 ± 5.0	66.2–82.1	0.214
	GPT-5	75.0 ± 8.4	61.6–88.4	
	Microsoft Copilot	78.4 ± 1.9	75.3–81.4	
	Google Gemini	69.3 ± 5.6	60.3–78.2	

* Upper bound truncated to 100 due to natural limit. # Using ANOVA test. Post hoc analysis using Tukey’s HSD test showed that copilot was better than Gemini in accuracy (*p* = 0.005). Also, GPT-5 showed higher accuracy than Gemini (*p* = 0.017). Regarding sensitivity, post hoc analysis showed no significant differences between the tools.

**Table 5:** Comparison of AI Model Reproducibility and Agreement Between the First and the Second Run.

AI Model	Language	Identical Outputs (*n*)	Reproducibility Index Between 1st and 2nd Run (%)	Round 1 vs. 2 Agreement		
				Cohen’s Kappa (κ)	Cohen’s Kappa (κ) 95% CI	*p*-Value
GPT-5	English	52	86.6%	0.783	0.654–0.912	<0.001
	Arabic	46	76.6%	0.645	0.496–0.794	<0.001
GPT-4o	English	47	78.3%	0.676	0.533–0.819	<0.001
	Arabic	50	83.3%	0.751	0.616–0.886	<0.001
Microsoft Copilot	English	54	90.0%	0.836	0.717–0.955	<0.001
	Arabic	53	88.3%	0.815	0.692–0.938	<0.001
Google Gemini	English	42	70.0%	0.567	0.414–0.720	<0.001
	Arabic	42	70.0%	0.573	0.416–0.730	<0.001

95% CI: 95% Confidence Interval. Reproducibility index: The proportion of identical responses across the two runs.

**Table 6:** Comparison of AI Model Reproducibility and Agreement Across Languages (English and Arabic) for the First Run.

AI Model	Identical Outputs (*n*)	Reproducibility Index Between English and Arabic for the 1st Run (%)	Intra-Language Agreement for the 1st Run		
			Cohen’s Kappa (κ)	Cohen’s Kappa (κ) 95% CI	*p*-Value
GPT-5	46	76.7%	0.650	0.501–0.799	<0.001
GPT-4o	41	68.3%	0.532	0.375–0.689	<0.001
Microsoft Copilot	49	81.7%	0.701	0.556–0.846	<0.001
Google Gemini	44	73.3%	0.610	0.457–0.763	<0.001

95% CI: 95% Confidence Interval. Reproducibility index: The proportion of identical responses across English and Arabic for each model.

## Data Availability

The data that support the findings of this study are available from [Rana K. Abu-Farha] upon reasonable request due to privacy/ethical restrictions.

## Supplementary Materials

The following supporting information can be downloaded at: https://www.mdpi.com/article/10.3390/jcm15010162/s1, Table S1: Cases Included in the Study (those containing an error are written in RED).

## Abbreviations

The following abbreviations are used in this manuscript:

ML	Machine Learning
AI	Artificial Intelligence
TP	True Positive
TN	True Negative
FP	False Positive
FN	False Negative
HSD	Honestly Significant Difference
ANOVA	One-way Analysis of Variance
κ	Cohen’s Kappa Statistic
