# Supplementary material for: Evaluating the Performance of AI Large Language Models in Detecting Pediatric Medication Errors Across Languages: A Comparative Study
Source: J Clin Med. 2025 Dec 25;15(1):162. doi: 10.3390/jcm15010162 (PMC12786879; doi:10.3390/jcm15010162)
Supplement: Supplementary file 1 [file jcm-15-00162-s001.zip › jcm-4058673-supplementary.pdf]

Table S1: Cases Included in the Study (those containing an error are written in RED)

System	Case
Respiratory	Case 1: A 2-year-old boy (13 kg) presented with barking cough and mild stridor, but no respiratory distress at rest. He was diagnosed with croup and prescribed 2 mg dexamethasone syrup.
Respiratory	Case 2: A 6-year-old girl (20 kg) was seen for persistent sneezing and nasal congestion. She was diagnosed with allergic rhinitis and prescribed fluticasone propionate nasal spray (50 mcg/spray) in each nostril twice daily.
Respiratory	Case 3: A 2-year-old boy (13 kg) with a history of wheezing during viral infections presented with increased wheezing and fever over two days, worsening at night. He was diagnosed with asthma exacerbation and prescribed albuterol inhaler, 1 puff every 3–4 hours via spacer/mask.
Respiratory	Case 4: A 16-year-old boy (55 kg) with a 4-week history of coughing spells and post-tussive vomiting was diagnosed with pertussis. He was prescribed azithromycin 500 mg orally once daily on day 1, then 250 mg orally once daily on days 2 to 5.
Respiratory	Case 5: A 4-year-old boy (16 kg) with atopic dermatitis had recurrent wheezing triggered by viral infections, smoke, and cat exposure, causing nighttime coughing and missed school. He was diagnosed with persistent asthma and prescribed inhaled corticosteroid (Pulmicort Flexhaler 90 mcg) twice a day.
Respiratory	Case 6: A 9-month-old girl (8.5 kg) presented with inspiratory stridor worsening with crying, but was alert and interactive. She was diagnosed with croup with stridor and prescribed 0.45 ml of 2.25% inhaled racemic epinephrine diluted to 3 mL with normal saline via jet nebulizer.
Respiratory	Case 7: A 6-month-old boy (7.5 kg) was brought in for persistent nasal congestion and cough, with no fever and feeding well. He was diagnosed with a viral upper respiratory infection and prescribed saline nasal drops (two or three drops in one nostril) when needed.
Respiratory	Case 8: A 2-year-old girl (12 kg) presented with fever and cough; chest radiography confirmed right lower lobe consolidation. She was diagnosed with bacterial pneumonia and was prescribed amoxicillin 540 mg IV every 12 hours.
Respiratory	Case 9: A 3-year-old girl (14 kg) was admitted to the ICU with respiratory distress and thick purulent tracheal secretions, diagnosed with bacterial tracheitis. She was prescribed nafcillin 700 mg every 6 hours.
Respiratory	Case 10: A 5-year-old girl (18 kg) with cystic fibrosis presented with fever, thick green sputum, and weight loss, admitted for pulmonary management. She was diagnosed with a pulmonary exacerbation and prescribed ceftazidime (900 mg IV every 8 hours) plus tobramycin (180 mg IV once daily).

Respiratory	Case 11: A 10-year-old child (32 kg) presented with intermittent wheezing triggered by exercise and cold air, with no nighttime symptoms or school absences. The patient was diagnosed with intermittent mild asthma and prescribed Xopenex inhaled β2-agonist every 3-4 hours.
Respiratory	Case 12: A 1-year-old female infant (9 kg) with history of prematurity (24 weeks gestation).During follow-up visit her physician orders palivizumab for prophylaxis against Respiratory Syncytial Virus at a dose of 135 mg administered intramuscularly once a month.
Respiratory	Case 13: A 13-year-old girl (45 kg) with fatigue, intermittent fever, and a persistent productive cough. Diagnosed with pulmonary tuberculosis confirmed by sputum. Started on a four-month regimen: daily isoniazid 300 mg, rifapentine 1200 mg, moxifloxacin 400 mg, and pyrazinamide 1500 mg.
Respiratory	Case 14: A preterm male infant (980 grams) diagnosed with bronchopulmonary dysplasia (BPD). Initiated caffeine citrate therapy: Loading dose: 10 mg/kg caffeine citrate IV; Maintenance dose: 5 mg/kg caffeine citrate IV or oral once daily.
Respiratory	Case 15: A 1-month-old full-term female infant (5 kg) with worsening paroxysmal coughing, cyanosis, and post-tussive emesis. PCR confirmed Bordetella pertussis. Hospitalized and started on clarithromycin 75 mg orally divided into 2 doses/day for 5 days.
Endocrine	Case 16: A 3-week-old newborn (4 kg) diagnosed with congenital hypothyroidism, and was started on thyroxine 25 mcg PO twice daily.
Endocrine	Case 17: A 3-year-old boy diagnosed with type 1 diabetes mellitus on MDI (glargine and aspart) recently shifted to an insulin pump (Medtronic 780g MiniMed), but continued using long-acting insulin glargine while connected to the pump.
Endocrine	Case 18: An 11-year-old female (35 kg) with genetically confirmed autosomal recessive hypophosphatemic rickets type 2 (ENPP1 mutation) was on classical treatment with active vitamin D (calcitriol 0.25 mcg q12hr) and phosphorus (200 mg q6hr).
Endocrine	Case 19: An 8-year-old male with type 1 diabetes was on insulin treatment with degludec every 8 hours subcutaneously and glulisine insulin three times daily subcutaneously.
Endocrine	Case 20: A 7-year-old male (25 kg) diagnosed with growth hormone deficiency was started on growth hormone treatment with Genotropin 0.8 mg/day subcutaneously.
Endocrine	Case 21: A 6-year-old girl (22 kg) diagnosed with central precocious puberty, with height on the 95th percentile and advanced bone age, was started on Decapeptyl IM 3.75 mg (triptorelin) every 6 months.
Endocrine	Case 22: A 5-year-old boy (19 kg, 110 cm) with multiple pituitary hormone deficiency was on thyroxine 50 mcg/day PO once daily, hydrocortisone 152 mg/day PO divided in 3 doses, and growth hormone 0.6 mg/day SC once daily.

Endocrine	Case 23: An 8-year-old girl (25 kg) she was on growth hormone 0.875 mg/day SC. She was recently diagnosed with Turner syndrome, so her doctor increased to 1.25 mg/day.
Endocrine	Case 24: A 2-month-old male (4.5 kg) diagnosed with classical congenital adrenal hyperplasia was started on hydrocortisone 3.5 mg PO once daily and fludrocortisone 0.1 mg PO twice daily.
Endocrine	Case 25: A 3-year-old male with primary adrenal insufficiency, on maintenance hydrocortisone treatment, was admitted to the hospital with acute gastroenteritis and started on IV hydrocortisone stress dose of 50 mg per m^2 per day in 4 divided doses.
Endocrine	Case 26: A neonate (3 kg) with recurrent congenital hypoglycemia, diagnosed with hyperinsulinism, was started on diazoxide 5 mg PO three times daily.
Endocrine	Case 27: A 1-year-old female (9 kg) with hyperinsulinism, with an initial octreotide dose of 5 mcg/kg/day without response, was titrated up to a maximum of 20 mcg/kg/day using a Medtronic pump at a basal rate of 7.5 mcg/hr.
Endocrine	Case 28: A 3-year-old male (10.5 kg) with genetically confirmed osteogenesis imperfecta and recurrent fractures was on pamidronate. Due to unavailability, he was shifted to Zoledronic Acid infusion 10.5 mg once every 6 months.
Endocrine	Case 29: A 3-year-old male with a gain-of-function mutation in fibroblast growth factor receptor 3 (FGFR3), with a history of decompression surgery for foramen magnum stenosis, was started on VOXZOGO® (vosoritide) 15.0 mcg/kg/day SC.
Endocrine	Case 30: A 10-year-old male (28 kg) with growth hormone deficiency, due to lack of adherence with daily injections, was shifted to long-acting growth hormone Ngenla (somatrogon) at a dose of 0.66 mg/kg/week. When somapacitan (Sogroya) became available, he continued with a similar dose of 0.66 mg/kg/week.
Neurology	Case 31: A 10-month-old infant, failing to thrive and globally developmentally delayed, is suspected to have a metabolic disorder. He presents with recurrent episodes of focal seizure. He was diagnosed with suspected metabolic disorder with recurrent focal seizures and was prescribed Sodium valproate at a maintenance dose of 30 mg/kg/day.
Neurology	Case 32: A 10-year-old boy was diagnosed with ADHD.Past medical history: episodes of fainting during exercise. Blood pressure was normal for age. ECG: Shows prolonged QT interval (QTc = 500 ms). He was diagnosed with ADHD and prolonged Q-T syndrome. Prescribed Ritalin 10 mg/day. In two divided doses before breakfast and before lunch
Neurology	Case 33: A 2-year-old child (13 kg) has had two febrile convulsions: one was simple and one was complex. His developmental milestones are age-appropriate, and family history is unremarkable. He was diagnosed with febrile seizures and was prescribed maintenance therapy with levetiracetam 200 mg orally twice daily.

Neurology	Case 34: A 6-year-old child (20 kg) presented with acute flaccid paralysis. After an appropriate work-up, he was diagnosed with Guillain-Barré Syndrome (GBS) and was prescribed Intravenous methylprednisolone 600 mg/day for 5 days.
Neurology	Case 35: A 6-year-old child (20 kg) presented with frequent episodes of staring and unresponsiveness. A hyperventilation test triggered typical episodes, supporting the diagnosis of childhood absence epilepsy. Ethosuximide was the preferred treatment, but due to its unavailability, the child was started on Depakine syrup 150 mg twice daily.
Neurology	Case 36: A 3-day-old term newborn, weighing 3.5 kg, was delivered via cesarean section after obstructed labor. At birth, he required vigorous resuscitation. He was admitted to the NICU with poor respiratory effort and generalized hypotonia. Today, the baby developed a focal seizure that began 5 minutes ago and is still ongoing. He was diagnosed with neonatal seizure and was prescribed intravenous Phenobarbital 70 mg as an initial loading dose.
Neurology	Case 37: A 3-year-old child (15 kg) with uncontrolled epilepsy is currently receiving valproate sodium 200 mg orally twice daily. Neuroimaging is unremarkable, and developmental milestones are age-appropriate. An EEG shows generalized epileptic activity. He was diagnosed with uncontrolled epilepsy and prescribed valproate sodium 200 mg orally twice daily. Due to inadequate seizure control, you decide to add levetiracetam.
Neurology	Case 38: A 4-year-old boy (17 kg) presented with unsteady gait and altered level of consciousness. CSF analysis revealed elevated protein, and a brain MRI showed findings consistent with Acute Disseminated Encephalomyelitis (ADEM). He was prescribed Intravenous methylprednisolone 200 mg/day for 5 days.
Neurology	Case 39: A 5-month-old infant presented with sudden clusters of brief jerks (flexion of trunk, arms, legs), especially after awakening. These lasted for a few seconds, repeated 10 times per day, and were associated with loss of social smile and poor eye contact. An EEG showed hypsarrhythmia. Brain MRI revealed cortical dysplasia. He was diagnosed with Infantile Spasms and prescribed ACTH intramuscular 15 IU/day.
Neurology	Case 40: A 2-month-old infant (5 kg) presents with fever, poor feeding, lethargy, focal seizures, bulging fontanelle. CSF PCR is positive for HSV-1. He was diagnosed with HSV-1 Encephalitis and prescribed IV acyclovir 20 mg/kg every 8 hours for 10 days.
Neurology	Case 41: A 4-month-old infant (4 kg) presented with infantile spasms. MRI brain showed subependymal nodules and cortical tubers. He was diagnosed with Tuberous Sclerosis Complex with Infantile Spasms and started on Sabril® 100 mg twice daily.
Neurology	Case 42: A 2-year-old previously healthy boy (12 kg) complains of repeated episodes of “fainting” over the past month. These typically occur when he is angry/frustrated, involving crying, breath-holding, cyanosis, and collapse, with spontaneous recovery within minutes. He was diagnosed with breath-holding spells and prescribed Iron supplement 40 mg elemental iron/day for 3 months.

Neurology	Case 43: A 7-year-old boy (24 kg) complains of frequent episodes of staring and decreased school performance, described as daydreaming for 5-10 seconds, with immediate resumption of activity. Family history notes a maternal cousin with epilepsy. Physical examination is unremarkable. He was diagnosed with childhood absence epilepsy and prescribed Ethosuximide 250 mg PO twice daily.
Neurology	Case 44: A 2-year-old child (11 kg) was brought to ER with a generalized tonic-clonic seizure that started 10 minutes prior. Airway was secured, but IV access could not be obtained immediately. He was diagnosed with status epilepticus and given rectal diazepam of 5 mg.
Neurology	Case 45: An 8-year-old girl (25 kg) presented with severe, burning pain, allodynia, and swelling in her left foot following a minor ankle sprain. Symptoms were disproportionate to the injury. She was diagnosed with Complex Regional Pain Syndrome (CRPS) Type I and prescribed Gabapentin 100 mg orally three times daily.
Infectious Diseases	Case 46: A 1-year-old boy (10 kg) presented with acute otitis media. He was prescribed amoxicillin 250 mg orally twice daily.
Infectious Diseases	Case 47: A 4-year-old boy (16 kg) presented with dysuria and frequency. Urinalysis suggested a urinary tract infection and urine culture taken. Then, he was prescribed trimethoprim-sulfamethoxazole (TMP-SMX) 5 mL (containing 40 mg TMP / 200 mg SMX per 5 mL) orally twice daily.
Infectious Diseases	Case 48: A 10-month-old girl (8 kg) developed impetigo on her face. She was prescribed mupirocin 2% ointment to apply to the affected areas three times daily for 7 days.
Infectious Diseases	Case 49: A 3-year-old boy (14 kg) with a history of recurrent skin infections presented with a localized cellulitis. He was prescribed clindamycin 150 mg orally every 8 hours.
Infectious Diseases	Case 50: A 6-month-old infant (7 kg) presented with fever, irritability, and bulging fontanelle. Suspecting bacterial meningitis, empiric treatment with intravenous ceftriaxone 175 mg twice daily, and vancomycin 105 mg every 6 hours were initiated.
Infectious Diseases	Case 51: A 9-year-old girl (30 kg) with recent travel history developed bloody diarrhea, abdominal cramps, and fever. Stool culture was positive for Salmonella non-typhi strains. She was prescribed azithromycin 300 mg orally once daily for day 1 then 150 mg for days 2-3.
Infectious Diseases	Case 52: A 14-year-old boy (60 kg) was diagnosed with Lyme disease (early localized, erythema migrans with mild systemic symptoms). He was prescribed doxycycline 100 mg orally once daily for 14 days.
Infectious Diseases	Case 53: A 5-year-old girl (18 kg) presented with symptoms concerning for influenza during peak flu season. Rapid influenza test was positive. She was prescribed oseltamivir (Tamiflu) 60 mg orally twice daily for 5 days.
Infectious Diseases	Case 54: A 5-year-old child (20 kg) was prescribed 200 mg amoxicillin for his throat bacterial infection every 12 hours.
Infectious Diseases	Case 55: A 10-year-old child who takes theophylline for his asthma presented to the hospital with atypical pneumonia infection, he was prescribed erythromycin.

Infectious Diseases	Case 56: A 6-year-old child presented to the hospital for her complicated skin infection, her mother mentioned that she has cancer, and she takes Zofran for her nausea of chemotherapy, the doctor prescribed Linezolid as an anti-bacterial for her infection.
Infectious Diseases	Case 57: A 2-year-old child presented to the ER with watery diarrhea that started 3 days ago, fever, and abdominal pain. The doctor found that he had elevated WBC and the patient was diagnosed with Clostridium difficile-associated diarrhea. The mother mentioned that the baby is allergic to penicillin. Later, the doctor prescribed oral vancomycin for his infection.
Infectious Diseases	Case 58: A 6-year-old girl (19 kg) with cerebral palsy admitted complaining of cough and chocking. Upon physical examination she found to have respiratory distress and chest X-ray showed aspiration pneumonia. She was prescribed IV Clindamycin 254 mg every 8 hours and IV ceftriaxone 950 mg once daily.
Infectious Diseases	Case 59: An 11-year-old boy (38 kg) was diagnosed with fungal meningitis. He was prescribed IV amphotericin B deoxycholate 27 mg day.
Infectious Diseases	Case 60: A 9-year-old child (30 kg) with leukemia presented with fever and neutropenia. He was prescribed cefepime 1500 mg IV every 8 hours.
